# The Additional Role of the 3-Vessels and Trachea View in Screening for Congenital Heart Disease

**DOI:** 10.3390/medicina58020262

**Published:** 2022-02-10

**Authors:** Roxana Gireadă, Demetra Socolov, Elena Mihălceanu, Roxana Matasariu, Alexandra Ursache, Mona Akad, Iuliana Bujor, Ioana Scripcariu, Radu Florin Popa, Răzvan Socolov

**Affiliations:** 1 Department of Obstetrics and Gynecology, University of Medicine and Pharmacy ‘Gr. T. Popa’, 700115 Iaşi, Romania; rox.gireada@gmail.com (R.G.); demetrasocolov@gmail.com (D.S.); emih2001@yahoo.com (E.M.); carpalecsandra@yahoo.com (A.U.); Akad.mona@yahoo.com (M.A.); bujor.iuliana@gmail.com (I.B.); rfpopa2008@yahoo.com (R.F.P.); SOCOLOV.razvan@gmail.com (R.S.); 2 Department of Obstetrics and Gynecology, Cuza Vodă Hospital, 700038 Iaşi, Romania; 3 Department of Obstetrics and Gynecology, Elena Doamna Hospital, 700398 Iaşi, Romania

**Keywords:** 3-vessels and trachea view, congenital heart disease, prenatal screening

## Abstract

*Background and Objectives*: Although frequent and associated with high mortality and morbidity rate, congenital heart disease (CHD) has a suboptimal prenatal detection rate, with significant variation according to the scanning protocol. The aim of this study was to evaluate the role of the 3-vessels and trachea view (3VT) in detecting CHD, with or without the use of Color Doppler, with an emphasis on major CHD. *Materials and Methods*: We performed a retrospective study on 1596 unselected pregnant patients presenting at 11–37 weeks of gestation for a routine anomaly scan. We selected all CHD cases, and we analyzed the performance of the 4-chamber (4C) and 3VT view in detecting CHD. *Results*: A total of 46 fetuses with CHD were identified, yielding a 2.86% overall incidence, and 0.87% for major CHD. Grayscale 4C detected 47.8% of all CHD, going up to 71.7% by adding grayscale 3VT, with no major CHD remaining undetected by combining grayscale 4C and 3VT. *Conclusions*: Grayscale 4C and 3VT views are effective in detecting major CHD, thus proving their utility even in a low resource setting.

## 1. Introduction

Congenital heart disease (CHD) affects 0.8% of the population, while the incidence of severe CHD goes up to 0.2% [1]. Prenatal detection of CHD is crucial for planning delivery in cases that need immediate surgical treatment [2], and it helps parents decide the course of the pregnancy, especially when genetic testing is involved [3].

Routine anatomy scans must follow the local/international guidelines, but there are great disparities between sonographers, from the allotted scan time to the anatomy checklist. The International Society of Ultrasound in Obstetrics and Gynecology (ISUOG) and the American Institute of Ultrasound in Medicine (AIUM) favor the sweep technique from the upper abdomen to the upper mediastinum, adding Color Doppler if possible [4,5]. However, the mandatory routine scan includes only situs, the 4-chamber view (4C), alongside the left and right ventricular outflow tracts (LVOT and RVOT) [4,5]. Although outflow tract inclusion increased prenatal CHD detection rate, this remains suboptimal and varies considerably according to the number of cardiac views [6].

The 3-vessels and trachea view (3VT) was introduced by Yagel as a complementary cardiac view to easily assess the aortic arch anomalies [7]. The 3VT is the most cephalad cardiac transverse view, demonstrating the convergence of the aortic arch with the ductus arteriosus (DA), which communicates with the pulmonary artery at its bifurcation, near the origin of the left pulmonary artery [7]. In the same plane, the trachea and the superior right vena cava can be seen at the right side of the transverse aorta [7]. Several anatomic landmarks can be assessed using 3VT: vessel number, alignment, arrangement, and size; trachea sidedness; and thymus size. A subjective caliber comparison between the transverse aorta and the pulmonary artery (especially toward their convergence) is sufficient to raise suspicion of an outflow tract anomaly, like aortic coarctation or pulmonary stenosis. By adding Color Doppler, we can evaluate the flow through the transverse aortic arch and the pulmonary artery/DA and, moving slightly more cephalad, we can demonstrate the normal course of the right subclavian artery and of the left brachiocephalic vein (LVBC) [7].

Although the 3VT view is deemed ‘desirable if technically feasible’ by both the ISUOG and AIUM screening guidelines [4,5], especially due to its utility in detecting outflow tract anomalies [8,9,10], it is mandatory only in diagnostic echocardiography [11,12]. Hopefully, recommendations will change with future guideline revisions. The ISUOG guidelines were the screening reference in Romania, but national guidelines were adopted in 2019 for the 1st trimester (levocardia, situs solitus +/− 4C, and 3VT Color Doppler) [13] and 2nd trimester (situs solitus, levocardia, 4C, LVOT, RVOT +/− 3 vessels, 3VT, and Color Doppler) [14], and in 2020 for the 3rd trimester (4C, 3VT +/− Color Doppler) [15].

This study aims to evaluate the additional role of 3VT in detecting CHD in an unselected Romanian population, with or without the use of Color Doppler, with an emphasis on CHD that could require immediate care after birth.

## 2. Materials and Methods

### 2.1. Study Population

**Scheme 1:**
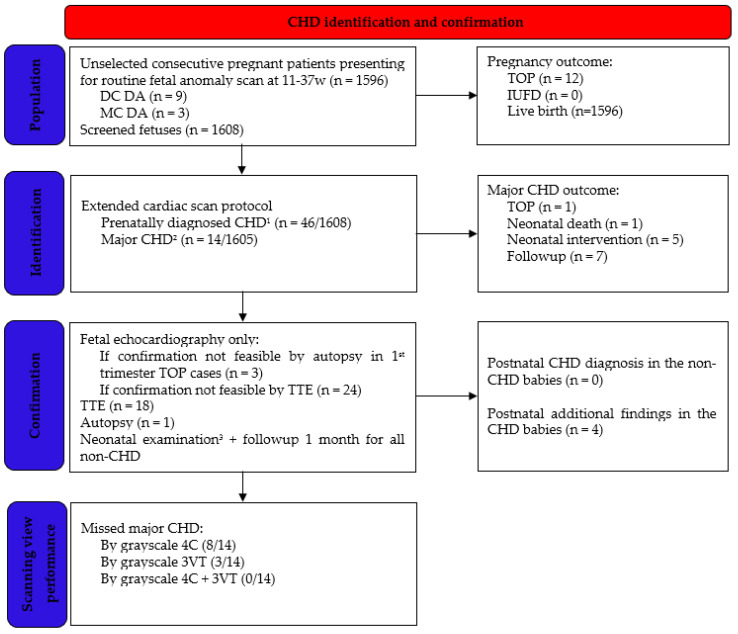
Flowchart clarifying the selection of study population. ^1^ Excluded CHD: cardiac rhythm disorders, persistent right umbilical vein, and umbilical vein varix. ^2^ Hygroma-associated CHD with early TOP was excluded from major CHD definition (n = 3). ^3^ General physical exam, heart auscultation, and preductal and postductal pulse oximetry. CHD, congenital heart disease; DC DA, dichorionic diamniotic twins; MC DA, monochorionic diamniotic twins; TOP, termination of pregnancy; IUFD, intrauterine fetal demise; TTE, transthoracic echocardiography; 4C, four chamber view; and 3VT, 3-vessels and trachea view.

This is a retrospective study conducted on unselected consecutive pregnant patients presenting at 11–37 weeks of gestation for a routine fetal anomaly scan in a private setting between 2019–2021. A total of 1608 fetuses were scanned (Scheme 1). We included only pregnancies with a known outcome that were scanned in their 2nd and/or 3rd trimester and pregnancies that were scanned only in the 1st trimester due to early termination for fetal anomaly. We collected data about demographics, ultrasound findings, prenatal genetic testing, and pregnancy outcome, by searching through the databases of the private clinics offering routine anomaly scans and of the hospitals where these patients gave birth, or by contacting patients via telephone or e-mail. We selected all cardiac/vascular anomalies detected by our scanning protocol, except for cardiac rhythm disorders, persistent right umbilical vein, and umbilical vein varix.

### 2.2. Ultrasound Examination

The scans were performed transabdominally ± transvaginally by three specifically trained sonographers, using a Voluson E8, S10 or E10 ultrasound machine (GE Healthcare, Milwaukee, WI, USA), RAB6-D/RAB6-RS/RAB7-C abdominal convex probe, 2–8 MHz, or a vaginal IC9-RS 3.6–10 MHz probe. The preferred gestational age for scanning was 11–13^+6^ weeks, 20–24 weeks, and 30–34 weeks.

In the 1st trimester, cardiac examination included situs, Color Doppler of the 4C and 3VT; the mandatory list also included the head (cranial vault, midline, cerebral ventricles, posterior fossa, facial profile with nasal bone, orbits with lenses, retronasal triangle, and mandibular gap), lungs, abdomen (diaphragm, stomach, kidneys, bladder, and abdominal wall), spine, limbs, and cord vessel number.

In the 2nd and 3rd trimesters, an extended cardiac protocol was followed using grayscale and Color Doppler, sweeping from abdominal situs to 4C (including Color Doppler examination of the atrioventricular septum in a horizontal orientation), left and right outflow tract, great vessels crossing, and 3VT (grayscale and Color Doppler, including a horizontal approach to further evaluate the supraaortic region for the course of the right subclavian artery and LVBC); aortic arch view, and bicaval view including identification of ductus venosus (DV). The routine anatomic survey for the 2nd and 3rd trimester also included the following elements: head (cranial vault, midline, cerebral ventricles, posterior fossa, corpus callosum, facial profile with nasal bone, orbits with lenses, lips, and nostrils), lungs, abdomen (diaphragm, stomach, gallbladder, intestine, kidneys, bladder, and abdominal wall), external genital organs, spine with cord insertion, limbs, and cord vessel number (the limbs and the abdominal wall were not mandatory in the 3rd trimester).

The allotted time for each patient was 45 min (75–90 min for twins), including history taking. The entire fetal anomaly scan lasted on average 35 min, while the cardiac examination itself took on average 10 min. Whenever protocol completion was not feasible due to inappropriate technical conditions, the patient was later rescanned. Upon fetal anomaly detection, a diagnostic ultrasound was performed by a fetal medicine specialist. Referral to a fetal cardiologist was made for all critical or ductal-dependent CHD, most likely requiring cardiac intervention shortly after birth, except for patients that opted for termination of pregnancy (TOP) after a 1st trimester diagnosis (in these cases, there was hygroma with multiple associated malformations).

### 2.3. Diagnosis Confirmation and Outcome Measures

The 1st trimester TOP CHD cases were confirmed only by transvaginal ultrasound performed by a fetal medicine specialist. All 2nd trimester TOP CHD were confirmed by autopsy.

All live-born babies (1596) were examined by the neonatologist in the first 3 days of life (general physical exam, heart auscultation, and preductal and postductal pulse oximetry). All children were followed up postnatally for 1 month.

All live-born babies with a prenatal CHD diagnosis underwent a transthoracic echocardiography, except for anomalies that could not be confirmed by this type of investigation, such as aberrant right subclavian artery (ARSA), persistent left superior vena cava (PLSVC), intrathymic LBCV, and DV agenesis. In addition, postnatal confirmation of atrial septal aneurysm (ASA) was not always possible due to its natural history toward physiological foramen ovale closure.

### 2.4. Outcome Measures

The complex/associated cardiac anomalies were classified according to the most severe or hemodynamically leading defect. CHD was considered major according to the possibility of requiring specialized care in the neonatal period. Under this spectrum, we decided to include all CHD with potential postnatal progression, such as mild pulmonary stenosis and PS; postnatal possible complications, such as extensive thrombosis from a DA aneurysm (DAA); and uncertain postnatal evolution, such as aortic coarctation. 1st trimester hygroma-associated CHD (3 cases) was excluded from the definition of major CHD, since parents usually opt for early TOP.

We evaluated the performance of the 4C and 3VT view ± Color Doppler in detecting all CHD and major CHD, respectively.

### 2.5. Statistical Analysis

We performed a descriptive statistical analysis using Excel (Microsoft Office 2019 Professional Plus, Microsoft Corporation, Redmond, WA, USA). Continuous variables were expressed as mean ± standard deviation. Scalar variables were expressed as median and range. Categorical variables were counted and expressed as percentages.

### 2.6. Ethical Approval

The study was approved by the Institutional Review Board of each clinic and hospital involved (75122/2021, 25/2021, 2176/2021, 15075/2021, 15611/2021, and 10771/2021).

## 3. Results

The study population included 1596 pregnancies, with a total of 1608 fetuses (nine twin dichorionic diamniotic pregnancies and three twin monochorionic diamniotic pregnancies). There were 12 TOP for fetal anomaly: six were terminated before 15 weeks (three for hygroma with multiple structural defects, including CHD), and six were terminated at 16–22 weeks (one for isolated severe aortic stenosis). Table 1 describes the demographics and pregnancy outcome of the 46 fetuses with prenatally diagnosed CHD (2.86% of the screened population).

Table 2 lists all CHD with their ultrasound findings and associated anomalies. Only 14/1605 (0.87%) were considered major CHD: 9/14 were detected due to 3VT—aortic coarctation/hypoplastic aortic arch/interrupted aortic arch (IAoA), mild PS, tetralogy of Fallot, DAA, and D-transposition; and 5/14 were detected with grayscale 4C—severe aortic stenosis, hypoplastic left heart syndrome (HLHS), pulmonary atresia with intact ventricular septum (PA/IVS), cardiac rhabdomyoma, and hypertrophic cardiomyopathy. There was no postnatal diagnostics of genetic anomaly in CHD live births.

Some CHD-associated defects were diagnosed only postnatally: aortic valve malformation, mild supravalvular PS + facial dysmorphism, perimembranous ventricular septal defect (VSD), and hypospadias. There was no cardiac anomaly diagnosed in the first month of life in the nonCHD population.

By analyzing the performance of the 4C and 3VT with and without Color Doppler, (Table 3), we can see that by using only grayscale 4C we detected 47.8% of CHD, and by adding grayscale 3VT we achieved a 71.7% detection rate. Adding Color Doppler to our examination increased the detection of small septal defects (provided the septum was evaluated horizontally) and of anatomic variants, such as ARSA and intrathymic LBCV.

Hereafter, we present several cases of CHD with a normal 4C view but detected due to grayscale and/or color 3VT.

**Figure 1:**
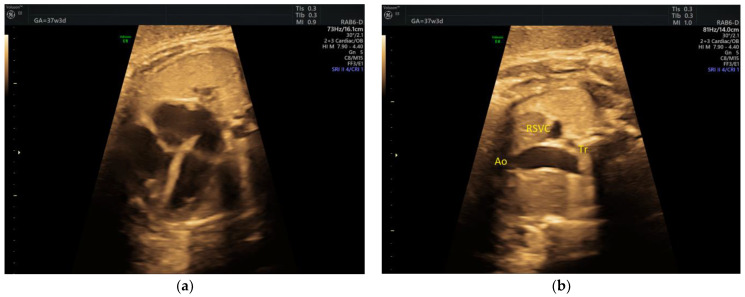
(**a**) D-transposition with normal 4C view; (**b**) Only two vessels (the aorta and the right superior vena cava) are seen on grayscale 3VT. Ao, aorta; RSVC, right superior vena cava; Tr, trachea (images from personal collection).

The D-transposition was detected in the 3rd trimester, after reportedly normally crossing great vessels at the 2nd trimester anomaly scan (Figure 1).

**Figure 2:**
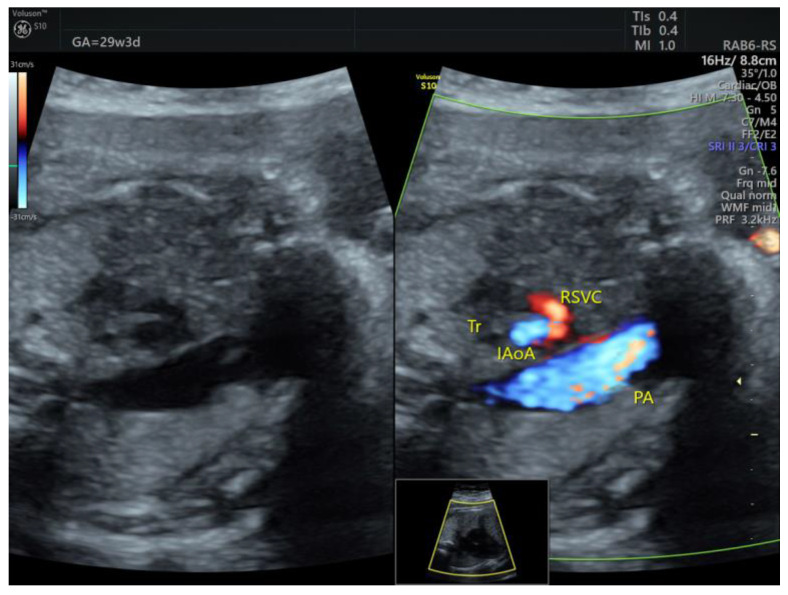
Abnormal 3VT in a case of interrupted aortic arch type B with associated malalignment VSD. RSVC, right superior vena cava; Tr, trachea; IAoA, interrupted aortic arch; PA, pulmonary artery (images from personal collection).

There was one case of interrupted aortic arch type B, with an associated malalignment VSD. Due to this association, the 4C seemed normal. On the 3-vessels view, the aorta was only slightly smaller than the pulmonary artery, but the ‘V’ was impossible to demonstrate on 3VT (Figure 2, Video S1).

**Figure 3:**
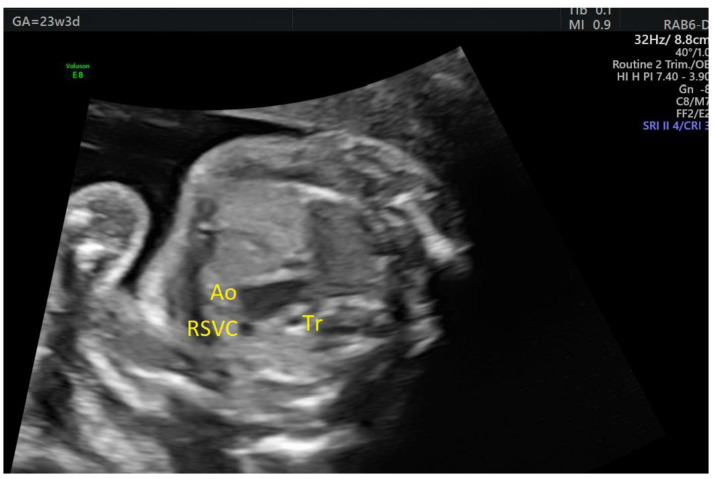
Abnormal 3VT in a case of tetralogy of Fallot: there is a large aorta, and the pulmonary artery cannot be identified. Ao, aorta; RSVC, right superior vena cava; Tr, trachea (images from personal collection).

In our tetralogy of Fallot case, there was also pulmonary atresia, so the 3VT was profoundly modified, with just a large aorta appearing instead of the ‘V’ (Figure 3).

**Figure 4:**
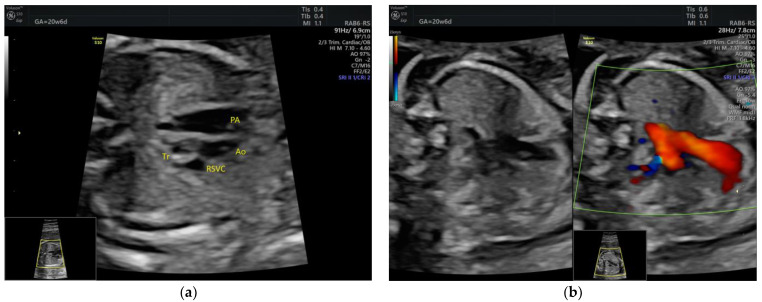
(**a**) Grayscale 3VT: the transverse aorta narrows gradually and is difficult to follow up to the ductus arteriosus; (**b**) Color 3VT: the aortopulmonary convergence appears normal, with great vessels seemingly having the same caliber. PA, pulmonary artery; Ao, aorta; RSCV, right superior vena cava; Tr, trachea (images from personal collection).

All cases of aortic coarctation were suspected due to a smaller transverse aortic arch on grayscale 3VT and confirmed by evaluating the aortic arch in a sagittal view. In one case, color 3VT seemed normal, but the transverse aorta was difficult to follow up to the DA (Figure 4). Sagittal examination of the aortic arch demonstrated a contraductal shelf (Video S2).

**Figure 5:**
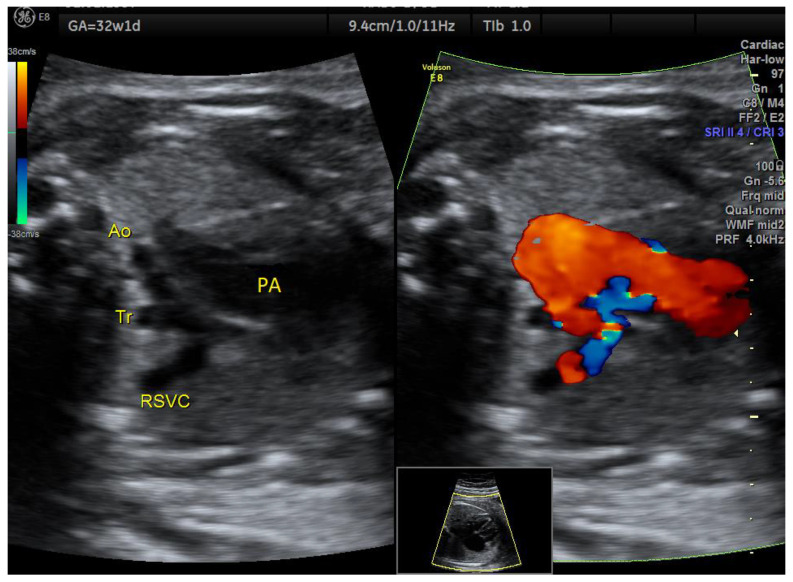
Atrial septal aneurysm at 32w1d: the transverse aortic arch is smaller than the pulmonary artery on 3VT, but with anterograde flow. Ao, aorta, Tr, trachea; RSVC, right superior vena cava; PA, pulmonary artery (images from personal collection).

**Figure 6:**
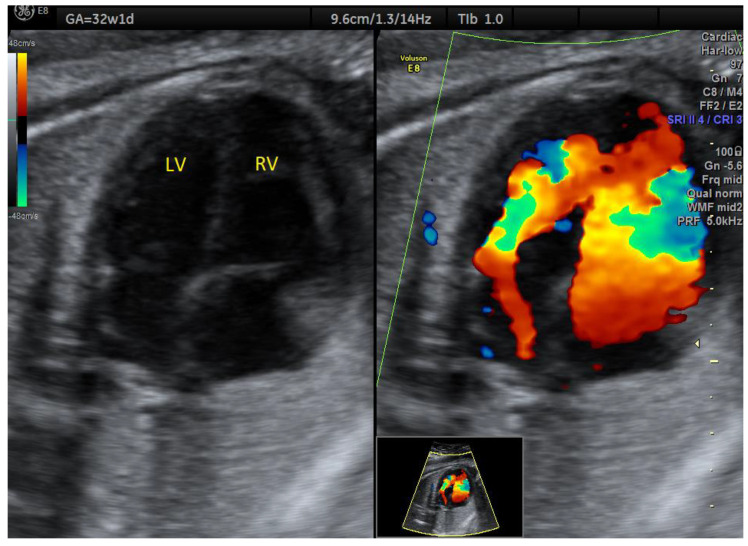
Atrial septal aneurysm at 32w1d: diminished left ventricular inflow. LV, left ventricle; RV, right ventricle (images from personal collection).

In one case of ASA detected in the 2nd trimester, aortic arch hypoplasia was suspected after evaluating the 3VT view in the 3rd trimester (Figure 5). There was anterograde flow through the transverse aortic arch, but Color Doppler examination of the 4C view showed that the aneurysm became partially obstructive of the left ventricular inflow (Figure 6, Videos S3 and S4). After birth, only a double atrial septal defect type II and a malformed nonstenotic aortic valve were found, so the cause of the smaller aortic arch was not certain.

**Figure 7:**
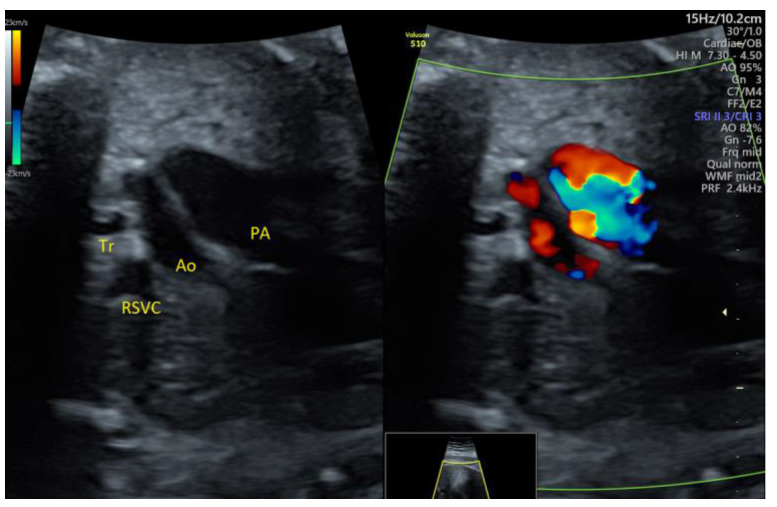
Mild pulmonary stenosis stable in the 3rd trimester: enlarged pulmonary artery with turbulent flow. PA, pulmonary artery; Ao, aorta; RSVC, right superior vena cava; Tr, trachea (images from personal collection).

We detected one case of mild PS in the 2nd trimester, based on the aortopulmonary discrepancy on grayscale 3VT (Figure 7, Video S5), which remained stable over time and had a good postnatal evolution.

**Figure 8:**
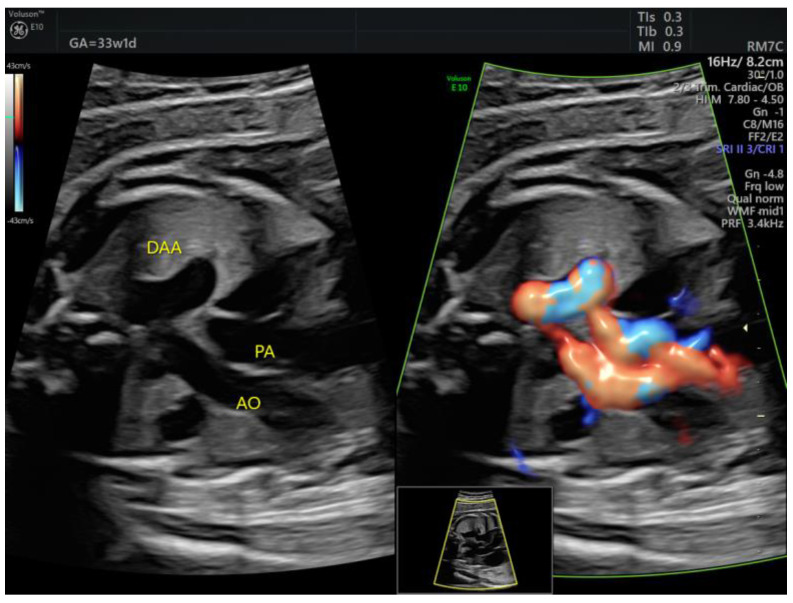
The ductus arteriosus forms a tortuous loop, which is evident on grayscale 3VT. DAA, ductus arteriosus aneurysm; PA, pulmonary artery; Ao, aorta (images from personal collection).

The DAA case diagnosed in the 3rd trimester (Figure 8, Video S6) had already presented a tortuous DA at 20w. There were no thrombotic complications before or after birth.

**Figure 9:**
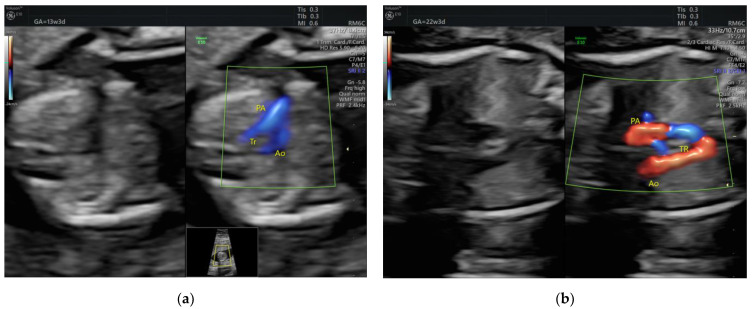
Right aortic arch with left ductus arteriosus showing its typical U shape on color 3VT (**a**) First trimester; (**b**) Second trimester. PA, pulmonary artery; Ao, aorta; Tr, trachea (images from personal collection).

The right aortic arch with left ductus arteriosus was detected at the 1st trimester scan (Figure 9), and Color Doppler proved essential for early identification of this anomaly.

**Figure 10:**
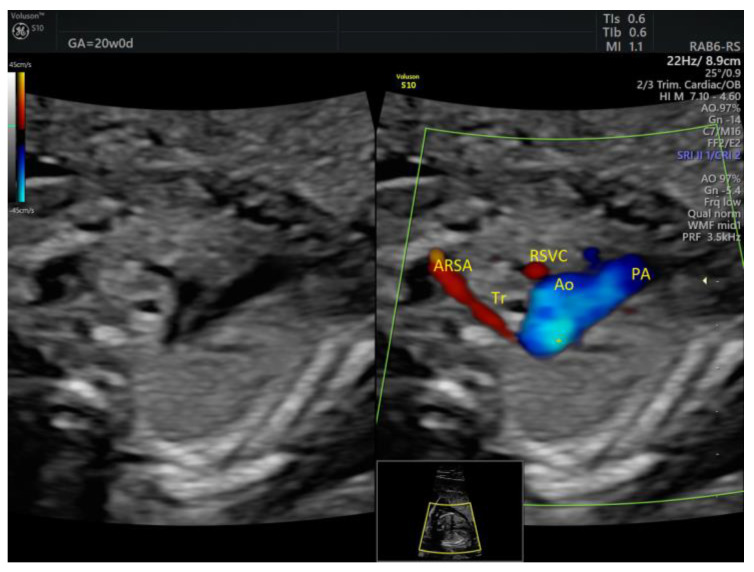
Aberrant right subclavian artery coursing behind the trachea at 20w0d. ARSA, aberrant right subclavian artery; Tr, trachea; RSVC, right superior vena cava; Ao, aorta; PA, pulmonary artery (images from personal collection).

ARSA can be identified by 3VT Color Doppler evaluation (Figure 10, Video S7). In our cohort, none of the prenatally isolated ARSA cases associated genetic anomalies, nor did they associate postnatal findings.

**Figure 11:**
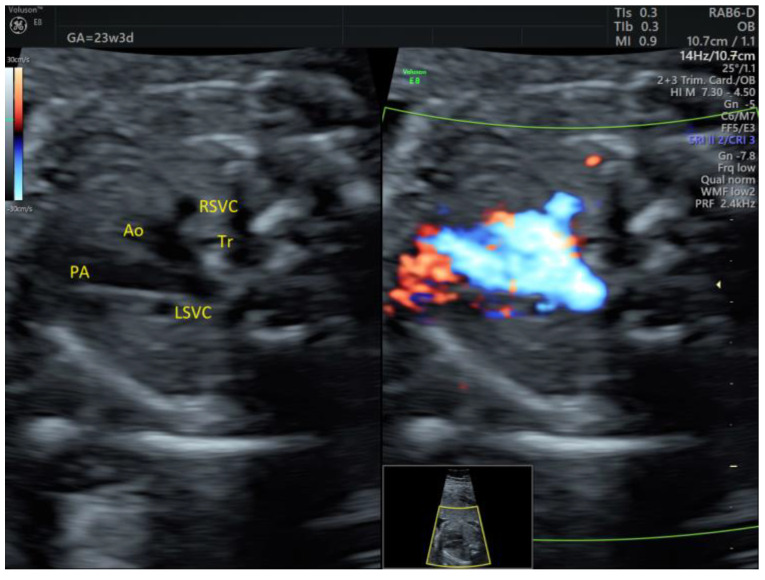
Persistent left superior vena cava is identified as a fourth vessel on the 3VT view, seen on the left side of the pulmonary artery. RSVC, right superior vena cava; Tr, trachea; LSVC, left superior vena cava; Ao, aorta; PA pulmonary artery (images from personal collection).

Color Doppler is also helpful in detecting PLSVC, although this can also be seen bordering the left atrium on a 4C view and on grayscale 3VT, it is easier detected upon failure to demonstrate a normal LBCV (Figure 11). None of the PLSVC cases from our cohort associated aortic coarctation or genetic anomalies.

## 4. Discussion

### 4.1. Comparison to Other Studies

The overall CHD incidence was higher when compared to other studies: 2.86% versus 0.8% [1], mostly because minor anomalies were included, such as PLSVC, ARSA, and ASA. Our study also found a higher major CHD incidence: 0.87% versus 0.2% [1]. That is partly because our definition of major CHD included some presumably nonsevere lesions, but potentially worsening after birth, such as mild PS [16,17].

Since its introduction by Yagel [7], the 3VT view has been extensively studied [9,10,18,19,20] and included in the routine anatomy scan by experienced sonographers, proving its utility time and time again. Thus, a meta-analysis by Liu et al. proved that detection increases from 58% when using 4C + LVOT + RVOT to 73.5% by adding 3VT [6]. Our study reports a comparable 3VT performance for detecting CHD (grayscale 4C + 3VT 71.7%).

### 4.2. Detecting Major CHD

3VT offers valuable clues leading to the diagnosis of both major and minor CHD. This study highlights the importance of 3VT in screening even without using Color Doppler. Just by adding grayscale 3VT examination to the 4C view, our study found that the detection rate increased 23.9%, with no major CHD being missed. It is noteworthy that the extra CHD detected by 3VT were outflow tract anomalies, whose outcome was significantly improved by prenatal diagnosis (D-transposition, coarctation of the aorta, and Fallot).

A simplified protocol scan using only grayscale 4C and 3VT could be employed during every scan, even when the referral reason is not an anatomic survey. This is especially useful for patients who never underwent an anatomy scan due to poor prenatal care access; for example, 78% of Romanian women underutilize free prenatal care [21]. There is also a ‘second opinion’ benefit, for evolving CHD, such as valvular stenosis, or for missed CHD due to incorrect/incomplete initial scans, such as the D-transposition diagnosed in the 3rd trimester after a reportedly normal 2nd trimester scan.

Thus, combining 4C + 3VT proved to be a powerful tool in a low resource setting, whether that resource is available scan time, appropriate sonography training, or technical challenges.

### 4.3. Detecting Minor CHD

Not only major CHD detection is of interest regarding perinatal mortality and morbidity, but also the detection of seemingly minor CHD, because of its potential evolution toward more serious CHD.

In fetal life, ASA is considered a normal evolution of an atrial septal defect toward spontaneous closure, so on postnatal echocardiograms it can present as normal, as a patent foramen ovale, or as an atrial septal defect [22]. Although generally considered a benign finding, on occasion ASA has been reported to become obstructive of the left ventricular inflow, with a consequent evolution toward mild left ventricle and aortic arch hypoplasia [23], so close follow up is advisable. This also happened in one of four ASA detected by our study in the 2nd trimester; fortunately, postnatal hemodynamic changes led to a nonobstructive ASA.

A tortuous DA is usually a minor ultrasound finding and considered a normal variant, but it is worth following up due to its potential evolution toward restrictive DA or DAA [24]. In our series, there was no restrictive DA, but a tortuous DA detected in the 2nd trimester developed into a DAA in the 3rd trimester. DAA can be associated with connective tissue disease [25] and can become complicated by prenatal thrombosis [26] or postnatal extension of the DA thrombus (whose formation is a physiologic event in order for the DA to close) to the adjacent pulmonary artery [27] or to the descending aorta [28]. Thus, an antenatal diagnosis of DAA ensures a proper follow up, with planned delivery in a specialized center where surgical treatment is available.

### 4.4. 3VT Advantages

Studying 4C and 3VT in grayscale is easy to learn and not time consuming. Because these are transverse views, one must just sweep cephalad from the upper abdomen, which is already routinely used to estimate fetal weight. In our opinion, under acceptable technical conditions, adding grayscale 4C and 3VT to a biometry scan would increase the examination time by maximum 1 min.

### 4.5. 3VT Pitfalls

There are some pitfalls in using 3VT in routine anatomy scans in a low-risk population. 3VT also detects normal variants, mostly being asymptomatic; however, they still increase parental anxiety, especially in ARSA or PLSVC cases when genetic anomalies are brought into question [29,30]. Genetic testing is questionable if the ultrasound marker is isolated; our study did not demonstrate ‘hidden’ anomalies for isolated ARSA/PLSVC.

Although reported as easy to obtain and to interpret [7], sometimes a ‘perfect V’ cannot be easily obtained; that is, the aortopulmonary convergence is difficult to demonstrate, as if the transverse aorta and the DA were not in the same plane, and this can mislead the inexperienced sonographer to false-positive findings. However, if their caliber is rather equal all the way up to the descending aorta, even if not obvious in the same plane, there is no abnormality; the reason is either an incidence artifact or a tortuous DA, which is more obvious and frequent in the 3rd trimester [31].

Moreover, 3VT increases the cost of CHD screening as reflected by increased screening time if technical conditions are difficult and increased referrals to maternal–fetal units. Increased referral is also stressful for the parents and puts an extra burden on services that provide diagnostic ultrasound; however, all significant CHD detection is worthwhile, whether it is syndromic or requiring specialized postnatal care.

Lastly, 3VT cannot completely replace LVOT and RVOT examination, since it does not directly evaluate the aortic and pulmonary valves, valvular stenosis being among the most common CHD [1]. As proven by the postnatal findings of this study, one mild PS and a malformed aortic valve were missed prenatally.

### 4.6. Strengths and Limitations

The strength of this study is the use of an extended scan protocol on an unselected population, thus detecting more cardiac and extracardiac anomalies than standard screening. Since most studies of extended cardiac examination performance are conducted on selected populations (either at risk for CHD or with suspicious screening results) [6], this study reflects more accurately the CHD frequency in an unselected fetal population. Also, some of these anomalies would be detected earlier according to our protocol. To our knowledge, this is the first study to report the routine use of 3VT in an unselected Romanian population.

There are several limitations to this study. There is an inclusion bias since private care is generally accessed by lower risk patients. Another study limitation is that children from the study population were followed up to 1 month, and there was no systematic postnatal echocardiographic examination, so there could be undetected CHD in our population. In our study, the scans were performed by experienced sonographers, who are very familiar with 3VT and go beyond the minimal guideline recommendations and routinely use the ISUOG recommended 5-planes sweep, with an allotted screening time longer than in public settings (45 min versus usually 30 min). Due to their experience in using 3VT (but also extra cardiac views), it is possible that that detection rates would be lower for inexperienced sonographers. Moreover, routine pulsed-wave Doppler was not used across the cardiac valves, so aortic/pulmonary stenosis may have been missed.

## 5. Conclusions

Fetal echocardiography can accurately diagnose most CHD, but it is not reasonable to expect experts to perform all screening scans. Ultimately, CHD detection relies on referral from screening sonographers, who apply simple and time-efficient protocols. For outflow tract anomalies, sonographer training is of utmost importance. A chain is as strong as its weakest link, so proper education of first-line sonographers is pivotal in improving prenatal CHD detection.

3VT proves ideal for CHD screening because it is fast to obtain, easy to learn, and sufficient to raise suspicion of outflow tract abnormality with subsequent referral to a specialist. In our opinion, grayscale 4C + 3VT is the perfect combination to screen for CHD: as shown by this study, no major anomaly would be missed by using this technique, even without the use of Color Doppler.

## Figures and Tables

**Table 1:** Characteristics of the study group.

Total, n = 46	Value
Maternal age	30 ± 4.85 y
Gravida	1 (1–4)
Nulliparity	67.3%
Male fetuses	60.4%
Syndromic/genetic	6.5% (3/46)
TOP	8.7% (4/46)
Livebirth	91.3% (42/46)
Neonatal death ^1^	2.1% (1/46)
Gestational age at birth	39 ± 1.2 w
Birthweight	3320 ± 497 g (2250–4300)
C-section (%)	71.7%

^1^ Hypoplastic left heart syndrome was approached with compassionate care.

**Table 2:** Defects in 46 fetuses antenatally diagnosed with cardiac heart disease.

Anomaly	Total	Associated, Prenatally ^1^	Associated, Postnatally ^1^	Main Suspicious View	Abnormal 3VT 2D	1st	2nd	3rd	Detected at First Presentation
ASD type II	2	Late FGR	1 hypospadias	4C	-	0	1	1	2/2
ASA	9	1 hypoplastic aortic arch in 3rd T	1 aortic valve malformation + double ASD type II	4C	−/+	0	4	5	7/9
VSD, muscular	2	-	-	4C color	-	0	0	2	0/2
VSD, perimembranous	2	2 × T18 (hygroma + multiple defects)	-	other	-	2	0	0	2/2
AVSD, complete	1	hygroma + multiple defects (no genetics)	-	4C	-	1	0	0	1/1
Hypertrophic cardiomyopathy	1	gestational diabetes	-	4C	-	0	0	1	1/1
Left cardiac axis deviation ^2^	1	echogenic CSP	mild supravalvular PS + facial dysmorphism	4C	+	0	1	0	1/1
Severe aortic stenosis	1	-	-	4C	+	0	1	0	0/1
Aortic coarctation	3	1 × (hypoplastic NB and late FGR)	-	3VT	+	0	2	1	2/3
IAoA	1	perimembranous VSD	-	3VT	+	0	1	0	1/1
HLHS	1	mitral atresia + aortic atresia	-	4C	+	0	1	0	1/1
Valvular PS, mild	1	-	-	3VT	+	0	1	0	1/1
PA/IVS	1	-	-	4C	+	0	0	1	1/1
Fallot	1	-	-	3VT	+	0	1	0	1/1
RAA	1	-	perimembranous VSD	3VT	+	1	0	0	1/1
D-transposition ^3^	1	-	-	3VT	+	0	0	1	1/1
Isolated ARSA	6	-	-	3VT color	-	0	6	0	2/5
PLSVC	4	-	-	3VT color	+	0	4	0	4/4
Intrathymic LBCV	3	-	-	3VT color	-	0	3	0	3/3
DAA	1	Tortuous DA at 20 w + late FGR	-	3VT	+	0	0	1	1/1
Cardiac tumors, *rhabdomyoma*	1	-	-	4C	-	0	1	0	1/1
DV agenesis	2	1 × (ARSA + bilateral pielectasis)	-	other	-	2	0	0	2/2

^1^ Structural and genetic defects. ^2^ Cardiac axis normalized by the late 2nd trimester. ^3^ The D-transposition had a reportedly normal 2nd trimester scan. ASD, atrial septal defect; FGR, fetal growth restriction; ASA, atrial septal aneurysm; VSD, ventricular septal defect; T18, trisomy 18; AVSD, atrioventricular septal defect; CSP, cavum septum pellucidum; PS, pulmonary stenosis; NB, nasal bone; IAoA, interrupted aortic arch; HLHS, hypoplastic left heart syndrome; PA/IVS, pulmonary atresia with intact ventricular septum; RAA, right aortic arch; ARSA, aberrant right subclavian artery; PLSVC, persistent left superior vena cava; LBCV, left brachiocephalic vein; DA, ductus arteriosus; DV, ductus venosus; 4C, 4-chamber; 3VT, 3-vessels and trachea.

**Table 3:** Prenatal ultrasound performance by scanning view.

View	Detected CHD, All n = 46	Missed CHD, Major ^1^ n = 14
Grayscale 4C only	47.8% (22/46)	57.1% (8/14)
Grayscale 3VT only	36.9% (17/46)	21.4% (3/14)
Color 3VT only	58.6% (27/46)	14.2% (2/14)
Grayscale 4C + 3VT	71.7% (33/46)	0% (0/14)

^1^ By using only grayscale 3VT, we would have missed severe aortic stenosis (at that particular stage), mild hypertrophic cardiomyopathy, and cardiac rhabdomyoma.

## Data Availability

The data used to support the findings of this study are available upon request to the corresponding author.

## Supplementary Materials

The following are available online at https://www.mdpi.com/article/10.3390/medicina58020262/s1: Video S1: Interrupted aortic arch and malalignment VSD; Video S2: Aortic coarctation—contraductal shelf; Video S3: Atrial septum aneurysm—a redundant foramen ovale flap that reaches the mitral valve; Video S4: Atrial septum aneurysm with diminished left ventricular inflow; Video S5: Mild pulmonary stenosis–dilated main pulmonary trunk; Video S6: Ductus arteriosus aneurysm—the tortuous DA can be followed leaving the pulmonary artery to reach the aorta; Video S7: ARSA (retrotracheally) and Right Common Carotid Artery (pretracheally).
